# Minimally invasive abdominal wall reconstruction with robotic-assisted transversus abdominis release following orthotopic liver transplant

**DOI:** 10.1007/s00464-026-13219-0

**Published:** 2026-08-05

**Authors:** Joe Banton, Jeffrey A. Blatnik, Robert MacGregor, Arnab Majumder

**Affiliations:** Section of Minimally Invasive Surgery, Department of Surgery, Washington University School of Medicine, 660 S Euclid Ave, Box 8109, St. Louis, MO USA

**Keywords:** Incisional hernia repair, Abdominal wall reconstruction, Robotic TAR, Liver transplant, Hernia

## Abstract

**Introduction:**

Incisional hernias following liver transplantation are common, particularly with bi-subcostal (Mercedes) incisions. Durable repair options and perioperative outcomes in this immunosuppressed population remain poorly defined. We analyzed outcomes following minimally invasive abdominal wall reconstruction (AWR) utilizing robotic-assisted transversus abdominis release (rTAR).

**Methods:**

Single-center, retrospective review of a prospectively maintained series of post-liver transplant patients referred for incisional hernia repair. Adult patients undergoing elective AWR via robotic TAR between January 2018 and July 2025 were included.

**Results:**

51 patients underwent elective hernia repair. Mean age was 61, mean BMI at repair was 29.7 kg/m^2^, and 65% were male. Mean time from transplant to repair was 20.8 months (range 7–64.9 mo). At time of repair, 27.5% were on single-agent, 58.8% on dual-agent, and 13.7% on triple-agent immunosuppression. Median hernia defect size was 144 cm^2^ (24–507 cm^2^) and median mesh size was 832 cm^2^ (390–1512 cm^2^). Mean operative time was 363 min and average length of stay was 3.8 days (2–9 days). Surgical site occurrences occurred in 4 patients (7.8%), including 2 surgical site infections. No reoperations were required. No hernia recurrences were identified at a median follow-up of 13.4 months.

**Conclusions:**

Robotic-assisted AWR with rTAR provides a durable and safe approach for complex post-transplant incisional hernias. Minimally invasive component separation limits wound morbidity in the setting of immunosuppression while enabling broad sublay mesh coverage. Further long-term follow-up is ongoing.

**Graphical abstract:**

**Figure:**
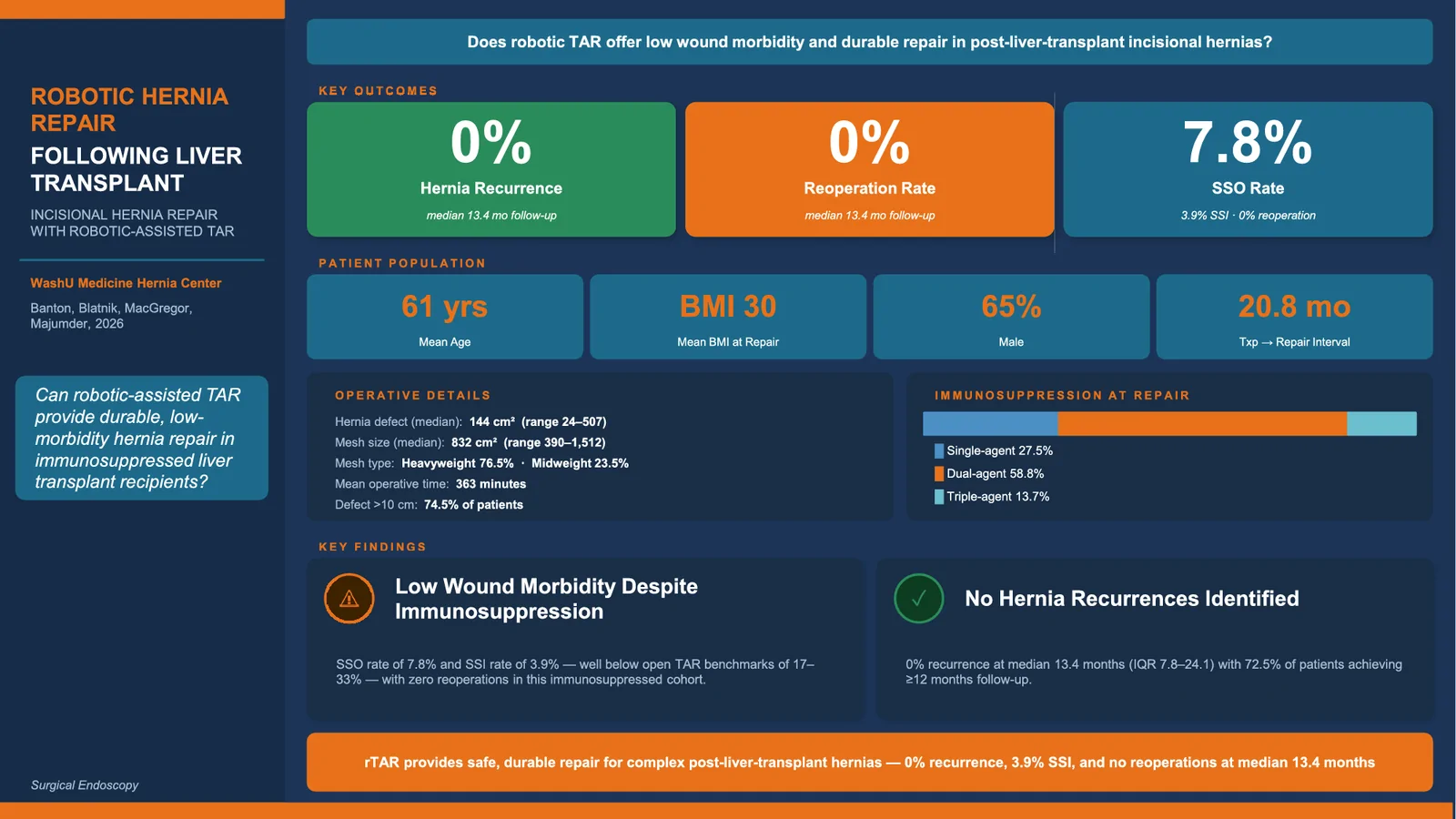

Orthotopic liver transplant (OLT) remains a lifesaving therapy for patients with end-stage liver disease with improving long-term survival [1]. As survival has increased with advances in surgical techniques and immunosuppressive regimens, more attention has been placed on long-term complications, including the development of incisional hernias. Incisional hernias following OLT occurs in up to 43% of liver transplant patients [2,3]. The most common operative technique for OLT employs a bilateral subcostal incision with midline extension, commonly called a Mercedes incision. These incisions are damaging to the muscle, fascial layers, and neurovascular supply of the anterior and lateral abdominal wall, frequently resulting in the development of complex and varied ventral incisional hernia defects.

Liver transplant recipients also represent a uniquely challenging population in the setting of abdominal wall reconstruction given the chronic immunosuppression that is required post-operatively. This immunosuppressed state leads to impaired wound healing, increased risk of surgical site infections, and further predisposes patients to high rates of hernia formation [4,5].

Multiple studies have demonstrated the safety and feasibility of the laparoscopic approach to hernia repair in the liver transplant population, with equivalent or improved rates of complication and recurrence in the laparoscopic groups as compared to open [6,7]. However, these study populations primarily reflected repair with intraperitoneal onlay mesh [8] which may have theoretical limitations—high rates of recurrence, implications in placement of intraperitoneal mesh—when addressing the destructive nature of the Mercedes incision.

Use of posterior component separation with transversus abdominis release (TAR) has been shown to be a safe and efficacious procedure to treat complex ventral hernias, and has since been applied across an increasingly broad spectrum of challenging reconstructive scenarios, including contaminated fields, recurrent repairs, and high-risk patient populations [9,10]. This technique enables more extensive myofascial advancement and offers broad sublay reinforcing mesh coverage. Abdominal wall reconstruction via an open TAR approach has demonstrated some reasonable durability, but the procedure is not without associated wound complications, particularly in these high-risk patients [11].

While performing transversus abdominis release is technically feasible via a laparoscopic approach [12], the da Vinci™ robotic platform has combined the durable benefits of posterior component separation (robot-assisted TAR, rTAR) and lower wound morbidity, with added dexterity and visualization. The ability to utilize this platform to provide extensive abdominal wall reconstruction though a minimally invasive approach is likely beneficial to such a challenging patient population, especially to reduce wound morbidity.

Despite widespread adoption of robotic-assisted abdominal wall reconstruction and rTAR, high-quality data remains limited when discussing its utilization in the unique post-liver transplant population. Given the anatomic complexity of hernias associated with the Mercedes incision and the impaired wound healing accompanying chronic immunosuppression, it is important to better define the safety and durability of robotic-assisted repair in this population. Our aim was to analyze our experience with robotic-assisted transversus abdominis release for repair of complex incisional hernias resulting from orthotopic liver transplant, focusing on perioperative outcomes, wound morbidity, and short-term durability.

## Methods

The study was approved by the Washington University Institutional Review Board (IRB). A retrospective review was performed to identify all patients who underwent OLT and subsequent rTAR between January 1st, 2018 and July 1st, 2025. All surgeons were fellowship-trained abdominal wall surgeons working in a tertiary referral center for hernias in a large academic institution.

Data reviewed and collected included patient demographic information such as age, sex, body mass index (BMI), race, smoking status, and comorbidities. Data related to liver transplantation were also gathered including operative details, postoperative complications, and immunosuppressive regimens. We also recorded hernia characteristics and operative details from hernia repair including type and size of mesh placed, and operative time. Postoperative outcomes included length of hospital stay, surgical site occurrences, surgical site infections, hernia recurrence, and duration of follow-up. Recurrence was determined via clinical examination unless interval cross-sectional imaging was available for review. Standardized cross-sectional imaging was not performed.

All statistical analyses were performed using Microsoft Excel (Microsoft Corporation, Redmond, WA) and ChatGPT 5.2 (OpenAI, San Francisco, CA). Categorical variables were compared using Chi Square test as applicable. Continuous variables were presented as means (standard deviation) or medians (range). For all comparisons, differences with a *p* value < 0.05 were considered statistically significant.

## Results

A total of 51 patients were identified who underwent AWR with robotic posterior component separation via TAR for repair of incisional hernias following orthotopic liver transplantation between January 2018 and July 2025 and included in the analysis.

### Patient demographics and clinical characteristics

Baseline demographic and clinical characteristics are summarized in Table 1. The cohort consisted of 33 male patients (65%) and 18 female patients (35%). The mean age at time of liver transplantation was 60 ± 9.5 years and the mean age at the time of definitive hernia repair was 61 ± 9.9 years. The mean interval between liver transplantation to hernia repair was 20.8 ± 13.1 months.

**Table 1:** Patient demographics and clinical characteristics

Characteristic	Value (*n* = 51)
Demographics	
Male sex, *n* (%)	33 (65%)
Female sex, *n* (%)	18 (35%)
Mean age at liver transplant (years)	60 ± 9.5
Mean age at hernia repair (years)	61 ± 9.9
Mean interval transplant to repair (months)	20.8 ± 13.1
Body mass index	
Mean BMI prior to transplant (kg/m^2^)	30.3 ± 6.4
Mean BMI at hernia repair (kg/m^2^)	29.7 ± 5.0
Smoking status	
Never smoker, n (%)	28 (54.9%)
Former smoker, n (%)	16 (31.4%)
Active smoker within 4 weeks of surgery, n (%)	7 (13.7%)
Prior Surgery	
Prior abdominal surgery in addition to transplant, *n* (%)	31 (60.8%)
Comorbidities	
Hypertension, *n* (%)	38 (74.5%)
Chronic kidney disease, *n* (%)	20 (39.2%)
Diabetes mellitus, *n* (%)	14 (27.5%)
Chronic obstructive pulmonary disease, *n* (%)	8 (15.7%)
Congestive heart failure, *n* (%)	6 (11.8%)

The mean body mass index (BMI) prior to liver transplantation was 30.3 ± 6.4 kg/m^2^, while the mean BMI at time of hernia repair was 29.7 ± 5.0 kg/m^2^. The majority of patients were never smokers, with 31.4% of patients being former smokers, and 13.7% were active smokers within four weeks of hernia surgery. A majority of patients (60.8%) had undergone prior abdominal surgery in addition to their liver transplantation. Common comorbidities included hypertension (74.5%), chronic kidney disease (39.2%), diabetes (27.5%), chronic obstructive pulmonary disease (15.7%), and congestive heart failure (11.8%).

### Liver transplant characteristics

Details related to the patients’ liver transplantation are presented in Table 2. The majority of patients underwent isolated OLT, while 3 patients underwent simultaneous liver-kidney transplantation. All liver transplant patients received a Mercedes type incision for access to the abdominal cavity.

**Table 2:** Liver transplant characteristics

Characteristic	Value (*n* = 51)
Transplant type	
Isolated orthotopic liver transplant (OLT), n (%)	48 (94.1%)
Simultaneous liver-kidney transplant, n (%)	3 (5.9%)
Fascial closure technique	
Running closure with #1 PDS™ suture, n (%)	50 (98.0%)
Interrupted closure alone, n (%)	1 (2.0%)
Post-transplant hospital course	
Mean hospital stay following transplant (days)	12.5 ± 12.8
Post-transplant complications	
Steroid-induced hyperglycemia, n (%)	23 (45.1%)
Return to OR, n (%)	7 (13.7%)
Port vein thrombosis, n (%)	3 (5.9%)
Other complications, n (%)	10 (19.6%)
Immunosuppression at time of hernia repair	
Tacrolimus (most common agent), *n* (%)	46 (90.2%)
Mycophenolate sodium, *n* (%)	33 (64.7%)
Everolimus, *n* (%)	4 (7.8%)
Cyclosporine, *n* (%)	3 (5.9%)
Prednisone at time of repair, *n* (%)	8 (15.7%)
Single-agent immunosuppression, *n* (%)	14 (27.5%)
Dual-agent immunosuppression, *n* (%)	30 (58.8%)
Triple-agent immunosuppression, *n* (%)	7 (13.7%)

Patients may be included in more than one immunosuppressive category due to combination therapy

Fascial closure during the transplant procedure was most commonly performed as a running technique with #1 polydioxanone (PDS™) suture (98.0%), with rare use of interrupted closure alone (2.0%). The mean length of hospital stay following transplantation was 12.5 ± 12.8 days.

Post-transplant complications were common with steroid-induced hyperglycemia occurring in nearly half the cohort. Seven patients (13.7%) required return to the operating room, most commonly for subcapsular hematoma evacuation. All patients remained on immunosuppressive therapy at the time of hernia repair. Tacrolimus was the most frequently used agent, followed by mycophenolate sodium, everolimus, and cyclosporine. Eight patients remained on prednisone at the time of hernia repair. Combination therapy was common, with 58.8% of patients on dual-agent therapy and 13.7% of patients on triple-agent therapy.

### Hernia repair perioperative details

All patients underwent rTAR with sublay mesh placement. Perioperative details are summarized in Table 3. Most patients presented with large hernia defects, with 38 patients (74.5%) having the largest hernia dimension greater than 10 cm. In four patients (7.8%), hernia dimensions were not recorded. Among the remaining 47 patients, the mean hernia size was 12.8 ± 6.4 cm (length) × 14.7 ± 7.1 cm (width). Mesh reinforcement was most commonly performed with Bard® Mesh, used in 38 cases (74.5%). Bard® Soft Mesh was used in 11 cases (21.6%), while Ethicon™ Prolene Mesh and Bard® Ventralight Mesh were each used in one case (2.0%). The mean operative time for the hernia repair was 362.9 ± 72.2 min.

**Table 3:** Hernia repair perioperative details

Perioperative variable	Value (*n* = 51)
Hernia defect characteristics	
Largest hernia dimension < 3 cm, *n* (%)	0 (0%)
Largest hernia dimension 3–10 cm, *n* (%)	9 (17.6%)
Largest hernia dimension > 10 cm, *n* (%)	38 (74.5%)
Hernia dimensions not recorded, *n* (%)	4 (7.8%)
Mean hernia length (cm) [*n* = 47]	12.8 ± 6.4
Mean hernia width (cm) [*n* = 47]	14.7 ± 7.1
Median hernia defect area (cm^2^)	144 (range 24–507)
Mesh type	
Heavyweight mesh, *n* (%)	39 (76.5%)
Bard® Mesh, *n* (%)	38 (74.5%)
Ethicon™ Prolene Mesh, *n* (%)	1 (2.0%)
Midweight mesh, *n* (%)	12 (23.5%)
Bard® Soft Mesh, *n* (%)	11 (21.6%)
Bard® Ventralight Mesh, *n* (%)	1 (2.0%)
Median implanted mesh size (cm^2^)	832 (range 390–1512)
Operative time	
Mean operative time (minutes)	362.9 ± 72.2

### Postoperative outcomes

Postoperative outcomes are summarized in Table 4. Mean hospital length of stay following hernia repair was 3.8 ± 1.5 days. Surgical site occurrences (SSO) were identified in 4 patients (7.8%), including 2 patients (3.9%) who developed surgical site infections (SSI). No patients required reoperation related to their hernia repair. At a median follow-up of 13.4 months (interquartile range 7.8–24.1 months), no hernia recurrences were identified in the cohort. 41 patients (80.4%) underwent clinical examination to determine recurrence while 10 (19.6%) had interval cross-sectional imaging available for review which did not demonstrate radiological recurrence.

**Table 4:** Postoperative outcomes

Outcome	Value (*n* = 51)
Hospital course	
Mean hospital length of stay (days)	3.8 ± 1.5
Range (days)	2–9
Wound / surgical site morbidity	
Surgical site occurrence (SSO), *n* (%)	4 (7.8%)
Surgical site infection (SSI), *n* (%)	2 (3.9%)
Reoperation related to hernia repair, *n* (%)	0 (0%)
Hernia recurrence	
Median follow-up (months)	13.4 (IQR 7.8–24.1)
Clinical follow-up, *n* (%)	41 (80.4%)
Radiological follow-up, *n* (%)	10 (19.6%)
Hernia recurrence, *n* (%)	0 (0%)

## Discussion

Incisional hernias are among the most common long-term complications following OLT, with reported rates ranging from 2 to 43% across previously published series [13]. The large Mercedes incision required in liver transplantation results in complex midline and off-midline abdominal wall defects that present a unique reconstructive challenge. The optimal surgical approach for incisional hernia repair in the post-liver transplant population is not well elucidated in the literature. Much of the existing literature is based on open and laparoscopic techniques with less than desirable recurrence rates. [2,3] The difficulty of any repair is further compounded by the lifelong immunosuppressive regimens required following transplantation, which impair wound healing through multiple mechanisms [4]. Large registry-based analyses—most notably the AHSQC series by Haskins et al.—have demonstrated that immunosuppression is not an independent predictor of 30-day surgical site infection or serious wound morbidity, however it does appear to be associated with higher rates of surgical site occurrences with limited data on long-term recurrence rates [14]. This underscores the importance of selecting a reconstructive strategy that minimizes wound burden while maximizing a durable repair, a balance that can be particularly difficult to achieve with conventional, open approaches in this population.

Posterior component separation via TAR has been shown to safely and effectively address complex, large-defect ventral hernias in multiple patient populations. Open TAR has demonstrated excellent durability, but the morbidity profile of the procedure, including SSO ranging from 17% to over 33% in prior studies, is of significant concern in immunosuppressed patients who are inherently vulnerable to wound breakdown and infectious complications [6, 15]. Tastaldi et al. addressed the use of open TAR in the post-OLT population, reporting outcomes of open TAR for complex incisional hernias following orthotopic liver transplantation using the AHSQC database. In their cohort of 44 patients, there was a 25% hernia recurrence rate at a median follow-up of 13 months, driven in large part by central mesh fractures. They also noted a 13.6% SSOPI rate, 11.3% SSI rate, and 7% reoperation rate. Their results highlight the possible limitation of open reconstruction in this population and the need for alternative approaches [11]. Against this backdrop, the adoption of minimally invasive platforms for posterior component separation has been of particular interest.

The robotic platform has enabled an adaptation of TAR that combines the anatomic advantages of posterior component separation with the wound morbidity advantages of a minimally invasive approach. Essentially, we are able to achieve myofascial advancement and create a large sublay space for mesh placement while, minimizing the total wound length / area generally needed to complete such an operation. Multiple comparative studies and meta-analyses have now confirmed that rTAR is associated with significantly lower overall complication rates and shorter hospital length of stay compared to open TAR, while achieving equivalent recurrence rates. Bracale et al., in a systematic review and meta-analysis of six studies comparing rTAR to open TAR in 831 patients, found that the robotic approach was associated with an overall complication rate of 9.3% versus 20.7% for open repair [12]. These results have been corroborated in subsequent multicenter comparisons including the study from Dewulf et al., which similarly demonstrated shorter hospital stay and fewer serious complications with rTAR, though at the expense of longer operative times [16]. More recently, a single-surgeon series by Turcotte et al. evaluating TAR in both open and robotic applications again found that rTAR led to fewer overall complications (11% vs. 35.7%) and shorter hospital length of stay (1d vs. 6d) [17]. Due to our extensive experience with minimally invasive abdominal wall reconstruction, we began to utilize this approach for our transplant patients.

The specific application of robotic abdominal wall reconstruction via TAR to post-transplant incisional hernia repair remains limited in the literature. The most directly comparable series to our own is a recent study from da Silveira et al., who evaluated robotic repair of post-transplant lateral incisional hernias across a mixed cohort of liver and kidney transplant recipients, with 83% undergoing TAR. In their series of 24 patients, 29.2% experienced surgical site occurrences with no hernia recurrences at a median follow-up of 11.2 months [18]. Our series, which focuses exclusively on liver transplant recipients with Mercedes incisions, revealed a 7.8% SSO rate, 3.9% SSI rate, and no recurrences at a comparable median follow-up of 13.4 months. We believe this reflects several compounding advantages of the robotic platform. The ability to obtain large retromuscular sublay mesh overlap is similar to the open operation despite use of a minimally invasive approach. In addition to the low wound morbidity, we note a significant improvement in the recurrence rate as compared to Tastaldi et al. Specifically, we believe the use of heavyweight mesh has reduced the rate of central mesh failures seen in other earlier series [11]. Our study includes a proportion of repairs with midweight mesh, but reflects our transition away from these products and we would advocate for the use of a heavyweight construct in this subset of patients given the considerable myofascial disruption that arises and persists following a Mercedes incision. This often leads to suboptimal myofascial coverage of the mesh and thus increases the strain on the mesh itself. The use of heavyweight mesh transfers more load on the reinforcement layer as compared to a lighter weight product. Further, the ability to perform this operation with six small incisions minimizes the opportunity for surgical site infections despite the use of immunosuppression.

The relationship between immunosuppression and wound morbidity in our cohort merits discussion. In our own experience, Kushner et al. evaluated the impact of immunosuppression, specifically on perioperative wound morbidity in patients undergoing TAR, and found that immunosuppression did not independently increase the rate of 30-day wound events among patients undergoing either open or robotic TAR [19]. However, this short-term picture does not necessarily lead to long-term success for these patients. Lee et al. demonstrated in a comparative analysis of immunocompromised and immunocompetent patients undergoing synthetic mesh hernia repair that while infection rates were equivalent, immunosuppression was independently associated with a more than threefold increase in the odds of hernia recurrence (OR 3.264, *p* = 0.012) [20]. More recently, Saidy et al. corroborated this finding in their analysis, demonstrating that the number of immunosuppressive agents a patient was receiving was a significant independent predictor of both postoperative complications and recurrence—suggesting a dose-dependent burden of immune compromise on long-term durability [21]. The relationship between repair and immunosuppression has been further supported by a large JAMA Surgery database study finding immunosuppressant use associated with increased odds of hernia recurrence across more than 15,000 patients with mesh repair [22].

Our results were encouraging as the majority of patients in our cohort were on combination immunosuppression at the time of repair, and despite this, wound morbidity remained low with less than 5% SSI rate seen in our cohort. This provides additional reassurance that rTAR, when performed with appropriate patient selection, can be safely executed in the transplant population without excess concern for surgical site occurrences attributable to immunosuppression alone.

Our experience is not without its limitations. This was a retrospective series with relatively short-term follow-up (median 13.4 months, IQR 7.8–24.1 months) with 72.5% achieving a minimum of 12 months follow-up, which may not capture late hernia recurrences that are known to occur in this population. Selection bias is inherent in those who were selected for robotic TAR for repair of their hernias, however this became the standard option as we gained experience with minimally invasive abdominal wall reconstruction. Our practice pattern in the transplant population slowly shifted toward the robotic approach over the past 5 years. It is not within the scope of this study to determine superiority over an open approach. Additionally, hernia recurrence was assessed through clinical follow-up rather than routine cross-sectional imaging, which may underestimate true recurrence rates, as subclinical or early recurrences may not be detectable on physical examination alone. Finally, our results may not be generalizable, as this study was conducted at a tertiary level hernia center with fellowship-trained surgeons and an extensive transplant program with excellent postoperative support.

Our experience with robotic-assisted abdominal wall reconstruction utilizing transversus abdominis release demonstrates a safe and durable method of hernia repair for the challenging and uniquely vulnerable post-liver transplant population. Our series demonstrated a minimal 3.9% SSI rate, no reoperations, and no hernia recurrences at a median follow-up of 13.4 months. The minimally invasive approach minimizes wound morbidity in patients where wound complications can lead to longer term consequences in immunosuppressed patients. Additionally, posterior component separation with wide sublay reinforcement permitted by TAR addresses the anatomic demands of complex hernias resulting from Mercedes incisions and the use of heavyweight mesh has essentially removed the incidence of central mesh fractures noted in prior series. Overall, we would advocate this approach to patients with complex hernias following liver transplantation.
